# Participation of Iranian Cerebral Palsy Children in Life Areas: A Systematic Review Article

**Published:** 2017

**Authors:** Marzieh PASHMDARFARD, Malek AMINI, Afsoon HASSANI MEHRABAN

**Affiliations:** 1 Department of Occupational Therapy, School of Paramedical and Health, Zanjan University of Medical Sciences, Zanjan,Iran.; 2 Department of Occupational Therapy, School of Rehabilitation Sciences, Iran University of edical Sciences, Tehran, Iran.; 3 Department of Occupational Therapy and Rehabilitation Research Center, School of Rehabilitation Sciences, Iran University of Medical Sciences, Tehran, Iran.

**Keywords:** Cerebral palsy, Participation, Occupations, Life area

## Abstract

**Objective:**

Cerebral palsy (CP) is the most common cause of chronic disability that restricts participation in areas of occupations for children. The main aim of rehabilitation is enhancement of their clients for participation in occupations. The aim of this study was to overview of the factors influencing the participations of children with CP in Iran.

**Materials & Methods:**

A systematic, evidence-based process (Duffy 2005) was used. For data gathering electronic databases including Google scholar and Iranian and foreigner famous journals in the fields of pediatrics, were used. The main key words for search were Activity of Daily Living (ADL), Instrumental Activity of Daily Living (IADL), play, leisure, work, rest/sleep, social participation, and education. All the papers of this study were about the factors influencing the participation of Iranian CP children during 2000-2016. Totally, 156 articles were found eligible as for Iranian CP children study, of which 100 articles were discarded. Because of repetitive and duplicability of some articles, 17 articles were removed as well.

**Results:**

The most studies about Iranian CP children participations in life areas were in the ADL area of participation (N=12), and the lowest articles were in the area in the field of: Work (N=2), play (N=2), and sleep/rest (N=2). Most of the occupational therapists do not focus on the all life areas.

**Conclusion:**

In Iran, many researchers do not pay attention to the participation of CP children. Many articles just paid attention to the sensory, motor or cognitive components of their clients.

## Introduction

Cerebral palsy (CP) is a general term used to describe motor function disorders, beginning early in life. This disability is the result of injury or dysfunction of the brain (1). This underlying brain lesion is not progressive and occurs in the early stages of brain development (2). The prevalence of CP in different countries varies between 6.0 to 9.5 cases per 1000 live births, but most statistics indicate that the prevalence is 2 cases per 1000 live births (3, 4). The prevalence of this disorder in Iran is 2.06 cases per 1000 live births (5).

CP causes or its risk factors are divided into several categories as pregnancy age under 20 years, birth weight below 2500 gr, the risk factors related to mother, factors related to pregnancy (Dislodge the placenta, twining) and fetal factors (Bradycardia, fetal malformation, poor fetal growth) are common causes of CP disorder (2, 6). 

According to the high prevalence of CP and disorders associated with that and eventually needs to follow disorders occur in people, knowing about their needs and try to decreasing their needs seems necessary. Knowing and understanding their demands, especially their expectation in the field of rehabilitation (occupational therapy) is very important (7). Among the rehabilitation members that provide the rehabilitation services to CP people, occupational therapists play an important role (7). The occupational therapists use different technics (neurodevelopmental, sensory integration), approaches to provide the best services to their clients (8, 9), however, they know the main aim of rehabilitation is participation of CP children in all areas of occupations or life area. The needs and demands of CP people include support and companionship from others, marriage, social acceptance, access to urban facilities, education and work, right to access supportive organizations, and right to access to medical and rehabilitation services (7). According to the Occupational Therapy Practice Framework (OTPF) of American Occupational Therapy Association (AOTA), these needs are defined as a participation in the occupations. International Classification of Functioning disability and health (ICF) defines participation as an involvement in life situations (10). The participations areas or occupation areas defined in OTPF are consisting of 8 areas: Activity Daily of Living (ADL), Instrumental Activity Daily of Living (IADL), work, play, leisure, education, rest/sleep/ social participation) (10). The definitions of these areas are as follows: 

**Activity Daily of Living (ADL):** Activities oriented toward taking care of one’s own body. These activities are fundamental to living in a social world; they enable basic survival and well-being (10). **Instrumental Activity Daily of Living (IADL): **Activities to support daily life within the home and community that often require more com¬plex interactions than those used in ADLs (10). 

**Rest/Sleep:** Activities related to obtaining restorative rest and sleep to support healthy, active engagement in other occupations (10).

**Education:** Activities needed for learning and participating in the educational environment (8). Work: Labor or exertion; to make, construct, manufacture, form, fashion, or shape objects; to organize, plan, or evaluate services or processes of living or governing; committed occupations performed with or without financial reward (10).

**Play:** Any spontaneous or organized activity that provides enjoyment, entertainment, amusement or diversion (10).

**Leisure:** Nonobligatory activity intrinsically motivated and engaged in during discretionary time, that is, time not committed to obligatory occupa¬tions such as work, self-care, or sleep (10). 

**Social Participation:** The interweaving of occupations to support desired engagement in community and family activities as well as those involving peers and friends, involvement in a subset of activities involve social situations with others and support social interdependence. Social participation can occur in person or through remote technologies such as telephone calls, computer interaction, and video conferencing (10). 

Since the main purpose of rehabilitation services especially occupational therapy in CP children is enabling and trying to achieve these children to the maximum level of independency and participating in life areas, therefore, this study was carried out to overview of the factors influencing the participations of children with CP in Iran.

## Materials & Methods

To performing this study, a systematic, evidence-based process (Duffy 2005) was used (11). For data gathering, the following sources were used:

**1. Electronic databases:** Medlin, PubMed, Google scholar, CINAHL, OVID Medline, CINAHL, Cochrane, ProQuest, Up to Date, Web of Science, OT search, OT direct, Pedro, SID, Magiran, IRAN MEDEX, MEDLIB and Iran doc.

**2. Iranian and foreigner famous journals in the fields of pediatrics as follows: **Iranian Journal of Pediatrics (IJP), Iranian Rehabilitation Journal (IRJ), Iranian Journal of Child Neurology (IJCN), Archive Physical Medicine and Rehabilitation, Developmental Medicine, Child Neurology, Physical and Occupational Therapy in Pediatrics, American Journal of Occupational Therapy. The key words used individually or in combination according to the MESH were as follows: Cerebral palsy (CP), participation, Iranian CP children, Activity of Daily Living(ADL), Instrumental Activity of Daily Living(IADL), play, leisure, work, rest/sleep, social participation, education, quality of life, occupational therapy, OT, physical therapy, rehabilitation, CP participation, OTPF, ICF, participation assessment, participation scale. The inclusion and exclusion criteria are categorized in Table 1.

**Table 1:** Inclusion and Exclusion Criteria’s

**Inclusion criteria**	**Exclusion criteria**
The articles of Iranian CP children participations	The language of articles other than Persian and English language
The articles about ADL, IADL, Work, leisure, play, rest/sleep, education , social participation of Iranian CP children	Articles of participation of children’s with other diagnosis instead of CP children
In access of abstracts or full text of articles	The articles related to the CP populations of other countries instead of Iran
The articles has been since 2000-2016	

## Results

**Fig 1:**
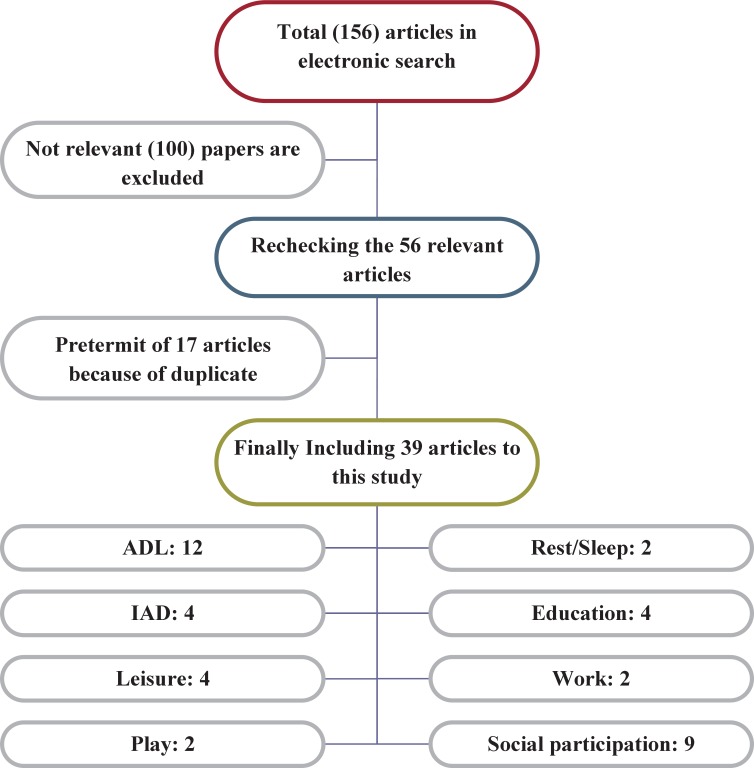
Results of systematic review search about participation of Iranian CP Children in different life area

According to the accessible databases, 156 articles were found in the scope of the study. Because of repetitive and duplicability of some articles, 17 articles were discarded. Thirty-nine articles were included (Figure 1). Some articles were consisting of more than one area of participation; therefore, these articles were categorized in more than one area. The results of all finding articles are presented in Table 2.

## Discussion

The aim of this study was to review the articles about Iranian CP children in different life areas. Participation promoting in different areas of occupations independently is the result and main outcome of rehabilitation services. Rehabilitation intervention in participating in life areas are more important that intervention in cognitive, motor, sensory, perceptual components (30). Furthermore, this systematic review helps to promote the rehabilitation interventions in the participations areas independently in contrast of rehabilitation interventions just on motor, sensory, or cognitive components. In this study, the most studies about Iranian CP children participations in life areas were in the ADL area of participation (N=12), and the lowest articles were in the area in the field of: Work (N=2), play (N=2), and sleep/rest (N=2). These findings can be inferred from the: 1) many of occupational therapist or other rehabilitation members, has not enough information about other life areas, many of them just know about ADL areas of participation; 2) maybe the parents of CP children do not know about the main aim of rehabilitation; 3) many of therapists do not know about occupation based practices; 4) the therapists do not know about the sufficient participation assessment tools. The most effective studies on the participation of Iranian CP children are as follows:

**1) Field of ADL: **The Bobath technique, Conductive Education, and education to parents, high level of GMFCS of CP Children, Occupational Therapy Home Program in the form of workshops and educational pamphlets with telephone follow-up, self-steam and self-confidence education, psychological rehabilitation of children with cerebral palsy, high-level of upper extremity function, physical fitness, enhancement of parents knowledge about how to take care of a child with CP professionally, spasticity reduction, appropriate handling can be mentioned as the facilitators of the participation of Iranian CP children in ADL and factors consisting: poor internal physical modification at home, poor practical training to family members and lack of assistive device can be mentioned as barriers of participation of Iranian cerebral palsy children in ADL (12-23).

**2) Field of IADL:** Minimizing barriers and providing more facilitators, appropriate handlings, right to access to housing, are the facilitators of the participation of Iranian cerebral palsy children in IADL. Participation diversity and intensity of CP children in the field of IADL are lower than their normal peers are (23-26).

**Table 2:** A Summary of the Methodology and Results of the Studies Used in This Study

	Authors	Title	Method	Life areas	Year	Results
1	Dalvand H , et al(12)	Effect of the Bobath Technique, Conductive Education and education to parent in Activities of Daily Living in children with CP	Quasi-experimental clinical trial with pre/post design	ADL	2009	The Bobath technique, CE, and education to parents improved ADL skills in children with cerebral palsy. But ,the most effective approach on ADLs of CP children was conductive education (CE), followed by education to parents and the Bobath technique
2	Nuranigharaborghe S, et al(13)	Relationship between Quality of Life and Gross Motor Function in Children with CP (Ages 4-12)	Cross – sectional study	ADL	2014	Gross motor functions of CP Children has an effective effect on adls area of CP children, in another word increase of gross motor function level of these children will improve their adls.
3	Afshar S, et al(14)	Effect of Occupational Therapy Home Program on Activities of Daily Living of 5-12 Yr Old Children	Randomized Clinical Trial (RCT)	ADL	2012	Occupational Therapy Home Program in the form of workshops and educational pamphlets with telephone follow-ups. Increased activities of daily living of children with CP and can be considered useful in addition to common occupational therapy programs.
4	Hosseini M A, et al (15)	The effect of self-steam and the level of self-care in CP children aged (11-15)	RCT	ADL	2002	Self-steam education will improve CP children ADLs skill especially self-care.
5	Razaviafzal Z, et al(16)	A Survey on caregivers' knowledge about special caring for 1-to-5 year-old children with CP and their compliance with these practices	Cross – sectional study	ADL	2013	The majority of the parents and caregiver had low to moderate levels of knowledge about how to take care of a child with CP professionally; caregivers should attend workshops and seek educational pamphlets to increase their knowledge about such methods of teaching toileting, mobility, carrying and sleeping techniques.
6	Rassafiani M, et al(17)	Hypertonicity in Children with Cerebral Palsy: a New Perspective	Review	ADL	2011	CP children experience problems in their all activities of daily living. Focusing on all aspects of the hypertonicity will surely help to decide better for these children and have better results.
7	Poursadoughi A, et al(18)	Psycho-Rehabilitation Method (Dohsa-hou) and Quality of Life in Children with Cerebral Palsy	Semi-experimental study with a pre-test - post-test design, follow-up and control group	ADL	2015	Psychological rehabilitation of children with CP improves their quality of life areas like: physical health, emotional wellbeing and self-esteem and…
8	Salehidehno N, et al(19)	Association between spasticity and the level of motor function with quality of life in community dwelling Iranian young adults With spastic cerebral palsy	Cross – sectional study	ADL	2012	Quality of life as a multi-dimensional concept has been impacted by many factors such as physical status, environmental issues and culture. Possibly, severity of spasticity and level of function have a less pronounced Effect on quality of life areas in adults with cerebral palsy.
9	Noori M, et al(20)	Relationship between upper extremity function and quality of life in the children with spastic CP in Tehran 2013	Cross-sectional study	ADL	2013	High level of upper extremity function is equal to higher level of quality of life areas and its scope. Therefore, in order to improve the upper extremity function, by programming and clinical reasoning, it is possible to promote the quality of life in spastic cerebral children.
10	Hamid Dalvand, et al(21)	Challenges in handling children with cerebral palsy: A qualitative content analysis	Qualitative research	ADL	2013	3 factors consist of: poor internal physical modification at home, poor practical training to family members and lack of assistive device leads to poor participation of cps in ADLs.
11	Balouchy R, et al (22)	Physical Fitness of The Children Infected With Cerebral Paralysis Through A work-group of "Therapists - Children - Parents"	RCT	ADL	2009	Physical fitness can improve the self-care level of CP children and can improve the confidence level of their parents.
12	Dalvand H, et al(23)	Handling in the Children with Cerebral Palsy: A Review of Ideas and Practices (A Literature Review)	Review	ADL	2012	Handling is the main component in the Occupational Therapy and the good handling can improve the ADL Skills of CP children and can improve the wellbeing level of parents and caregivers.
13	Hassani M, et al(24)	Enjoyment of participation in formal and informal Activities among students with CP and healthy students	Cross-sectional study	IADL	2015	CP had no effect on enjoyment of doing activities but could affect the participation diversity and intensity of children in formal and informal activities(IADL, Play, leisure, social participation)
14	Nobakht Z, et al(25)	Influence of child's disability on encountering environmental barriers to Participation of children with cerebral palsy	Cross – sectional study	IADL	2013	Minimizing barriersand providing more facilitators will improve CP children IADL.
15	Ghasemzadehr,et al(26)	Accessibility to the public facilities: a mean to achieve civil rights of The people with disabilities in Iran	Review	IADL	2008	The results of this study has categorized in6 fields: 1-**right to access to housing**,2- right to access to education and information, 3-right to access to job facilities, 4-right to access to medical care and rehabilitation,5- right to access to rest, leisure and sport and 6- right to access to places and transportation system
16	Dalvand H, et al(23)	Handling in the Children with Cerebral Palsy: A Review of Ideas and Practices (A Literature Review)	Review	IADL	2012	Handling is the main component in the Occupational Therapy and the good handling can improve the IADL Skills of CP children.
17	Hassani M, et al(24)	Enjoyment of participation in formal and informal Activities among students with CP and healthy students	Cross-sectional study	Play	2015	CP had no effect on enjoyment of doing activities but could affect the participation diversity and intensity of children in formal and informal activities(IADL, Play, leisure, social participation)
18	Ghasemzadehr,et al(26)	Accessibility to the public facilities: a mean to achieve civil rights of The people with disabilities in Iran	Review	Play	2008	The results of this study has categorized in6 fields: 1-right to access to housing,2- right to access to education and information, 3-right to access to job facilities, 4-right to access to medical care and rehabilitation,**5- right to access to rest, leisure and sport and** 6- right to access to places and transportation system
19	Hassani M, et al(24)	Enjoyment of participation in formal and informal Activities among students with CP and healthy students	Cross-sectional study	Leisure	2015	CP had no effect on enjoyment of doing activities but could affect the participation diversity and intensity of children in formal and informal activities(IADL, Play, leisure, social participation)
20	Ghasemzadehr,et al(26)	Accessibility to the public facilities: a mean to achieve civil rights of The people with disabilities in Iran	Review	Leisure	2008	The results of this study has categorized in6 fields: 1-right to access to housing,2- right to access to education and information, 3-right to access to job facilities, 4-right to access to medical care and rehabilitation,5-** right to access to rest, leisure and sport and** 6- right to access to places and transportation system
21	Pourranjbar M, et al(27)	Barriers for Wheelchair-User Disabled People to Participate in Leisure Physical Activities in Southeast of Iran	Cross-sectional study	Leisure	2015	Individual (job, physical fitness, economic and…), structural (Accessibility to the public facilities and…) and socio cultural (society, medias, families and…) barriers are the most important barriers that influence the leisure activities of disabled people.
22	Hassani Mehrabn, et al(28)	Comparison of participation between children with cerebralpalsy and typically developing peers 8-14 yr	Cross-sectional study	Leisure		There were significant differences between children with CP and typically developing peers in diversity and intensity of leisure activities but the enjoyment of leisure activities are same in both groups. Physical disability can affect participation of children in leisure activities.
23	Ghasemzadehr,et al(26)	Accessibility to the public facilities: a mean to achieve civil rights of The people with disabilities in Iran	Review	Rest/Sleep	2008	The results of this study has categorized in6 fields: 1-right to access to housing,2- right to access to education and information, 3-right to access to job facilities, 4-right to access to medical care and rehabilitation,**5- right to access to rest, leisure and sport and** 6- right to access to places and transportation system
24	Razaviafzal Z, et al(16)	A Survey on caregivers' knowledge about special caring for 1-to-5 year-old children with CP and their compliance with these practices	Cross – sectional study	Sleep/Rest	2013	The majority of the parents and caregiver had low to moderate levels of knowledge about how to take care of a child with CP professionally; caregivers should attend workshops and seek educational pamphlets to increase their knowledge about such methods of teaching toileting, mobility, carrying and sleeping techniques.
25	Hamid Dalvand, et al(21)	Challenges in handling children with cerebral palsy: A qualitative content analysis	Qualitative research	Education	2013	Poor handling at school because of: Inappropriate physical structure, poor attention to education and Inappropriate handling in school, lead to Inappropriate education of CP children.
26	Nobakht Z, et al(25)	Influence of child's disability on encountering environmental barriers to Participation of children with cerebral palsy	Cross – sectional study	Education	2013	Minimizing barriers and providing more facilitators will improve CP children education.
27	Ghasemzadehr,et al(26)	Accessibility to the public facilities: a mean to achieve civil rights of The people with disabilities in Iran	Review	Education	2008	The results of this study has categorized in6 fields: 1-right to access to housing,**2- right to access to education and information**, 3-right to access to job facilities, 4-right to access to medical care and rehabilitation,5- right to access to rest, leisure and sport and 6- right to access to places and transportation system
28	Nobakht Z, et al(29)	Environmental barriers to social participation of children with CP in Tehran		Education	2013	Environmental barriers in two groups : 1: Policies (services in community, policies of businesses , policies of education, policies of government ,) 2: physical and structural (design of home ,design of school, design of community , natural environment, Surroundings, technology), leads to poor education of CP children.
29	Ghasemzadehr,et al(26)	Accessibility to the public facilities: a mean to achieve civil rights of The people with disabilities in Iran	Review	Work	2008	The results of this study has categorized in6 fields: 1-right to access to housing,2- right to access to education and information, 3-**right to access to job facilities**, 4-right to access to medical care and rehabilitation,5- right to access to rest, leisure and sport and 6- right to access to places and transportation system
30	Nobakht Z, et al(25)	Influence of child's disability on encountering environmental barriers to Participation of children with cerebral palsy	Cross – sectional study	Work	2013	Minimizing barriers and providing more facilitators will improve CP children work.
31	Hassani M, et al(24)	Enjoyment of participation in formal and informal Activities among students with CP and healthy students	Cross-sectional study	Social Participation	2015	CP had no effect on enjoyment of doing activities but could affect the participation diversity and intensity of children in formal and informal activities(IADL, Play, leisure, social participation)
32	Hamid Dalvand, et al(21)	Challenges in handling children with cerebral palsy: A qualitative content analysis	Qualitative research	Social Participation	2013	Poor handling at society consist of: Psychological and religious abuse of CP children, inadequate government’s supports and inadequate civil facilities for CP children lead to Inappropriate Social Participation of CP children.
33	Nuranigharaborghe S, et al(13)	Relationship between Quality of Life and Gross Motor Function in Children with CP (Ages 4-12)	Cross – sectional study	Social Participation	2014	Gross motor functions of CP Children has an effective effect on Social area of CP children, in another word increase of gross motor function level of these children will improve their social participations.
34	Ghasemzadehr,et al(26)	Accessibility to the public facilities: a mean to achieve civil rights of The people with disabilities in Iran	Review	Social Participation	2008	The results of this study has categorized in6 fields: 1-right to access to housing,2- right to access to education and information, 3-right to access to job facilities, 4-**right to access to medical care and rehabilitation**,5- right to access to rest, leisure and sport and 6- **right to access to places and transportation system**
35	Nobakht Z, et al(29)	Environmental barriers to social participation of children with CP in Tehran	Cross-sectional study	Social Participation	2013	Environmental barriers in two groups : 1: Policies (services in community, policies of businesses , policies of education, policies of government ,) 2: physical and structural (design of home ,design of school, design of community , natural environment, Surroundings, technology), leads to poor social participation of CP children.
36	Poursadoughi A, et al(27)	Psycho-Rehabilitation Method (Dohsa-hou) and Quality of Life in Children with Cerebral Palsy	Semi-experimental study with a pre-test - post-test design, follow-up and control group	Social Participation	2015	Psychological rehabilitation of children with CP improves their quality of life areas like: social well-being, participation, access to service, and family health.
37	Abbaskhanian A, et al(30)	Rehabilitation Interventions for Children With Cerebral Palsy: A Systematic Review	Review	Social participation	2015	One of the main outcomes of Rehabilitation intervention is social participation, but in many interventions the therapists don’t pay attention to this outcome.
38	Salehidehno N, et al(19)	Association between spasticity and the level of motor function with quality of life in community dwelling Iranian young adults With spastic cerebral palsy	Cross – sectional study	Social Participation	2012	Quality of life as a multi-dimensional concept has been impacted by many factors such as physical status, environmental issues and culture. Possibly, severity of spasticity and level of function have a less pronouncedeffect on quality of life areas in adults with cerebral palsy.
39	Noori M, et al (20)	Relationship between upper extremity function and quality of life in the children with spastic CP in Tehran 2013	Cross-sectional study	Social Participation	2013	High level of upper extremity function is equal to higher level of quality of life areas and its scope. Therefore, in order to improve the upper extremity function, by programming and clinical reasoning, it is possible to promote the quality of life in spastic cerebral children.

**3) Field of Play:** Participation diversity and intensity of CP children in the field of play are lower than their normal peers, and right to accesses to play and sport can promote their participation in the field of play (24, 26) 

**4) Field of leisure:** The participation enjoyments of CP children in leisure activities are lower than other children, and factors such as: lack of access and benefits of facilities to participate in leisure activities, individual (job, physical fitness, economic), structural (Accessibility to the public facilities) and socio cultural (society, medias, families), are the effective barriers to participation in leisure activities (24, 26-28).

**5) Field of rest/Sleep:** the factors such as: enhancement of parent’s knowledge about how to take care of a child with CP professionally and right to assess and benefits of facilities to participate in sleeping and rest activities of CP children had effective influence on participation of CP children in the field of rest/sleep (16, 26).

**6) Field of Education:** The factors such as: poor handling at school because of inappropriate physical structure, poor attention to education and environmental barriers in two groups: 1) policies (services in community, businesses, education, government); 2) physical and structural (design of home, school, community, natural environment, surroundings, technology), leads to poor education of CP children and minimizing barriers and providing more facilitators to access to education and information improves the participation of education(21, 25, 26, 29).

**7) Field of Work:** Minimizing barriers and providing more facilitators to access to job leads to work participation (25, 26).

**8) Field of social participation:** The participation enjoyments of CP children in the field of social participation activities are lower than other children are. Factors such as: 1) lack of access and benefits of facilities, individual (job, physical fitness, economic), structural (Accessibility to the public facilities) and socio cultural (society, medias, families) barriers; 2) poor handling at society consist of: psychological and religious abuse of CP children, inadequate government’s supports and inadequate civil facilities for CP children; 3) poor attention of rehabilitation members team, and; 4) environmental barriers in two groups: 1) policies (services in community, businesses, education, government); 2) physical and structural (home, school, community, natural environment, surroundings, technology), leads to poor social participation of CP children the factors such as; 5) improvement in gross motor functions of CP children; 6) right to access to medical care and rehabilitation and right to access to places and transportation system; 7) psychological rehabilitation of children with cerebral palsy; 8) high level of upper extremity function; and 9) spasticity reduction leads to improvement of social participation activities of CP children (13, 19-21, 24, 26, 27, 29, 30). The good rehabilitation intervention needs good and appropriate assessment. Participation in the 8 areas is the main outcome of rehabilitation intervention especially in CP children (30). We used the culture based assessments tools. In Iran we have one assessment tool developed by Amini et al. especially, for assessment of Iranian children participation aged 6-12 yr. It has two versions: parent - report and child – report, this scale can assess the 8 areas of occupations and was developed based on OTPF (31). Another scale is CPQ (Children Participation Questionnaire), this questionnaire has been developed by Rozenberg et al. and can be assessed the 6 areas of occupation for children aged 4-6 yr (32), the psychometric properties of this questionnaire among Iranian children assessed by Amini et al., it has good psychometric properties for participation assessment of children aged 4-6 yr (33). Another important questionnaire is CP Quality of Life Questionnaire (CP QOL-Child), the psychometric properties of that, has been assessed by Soleimni et al. (34).

**In conclusion**, the main outcome of rehabilitation services is participation of CP children in all areas of occupations. According to OTPF, we have 8 dimensions in occupation (ADL, IADL, Work, Play, Leisure, Education, Rest/Sleep/ Social Participation) that all of them are important in enhancement of quality of life of CP children. None of them is preferred over another. According to that for good intervention in participation of CP children, using good and properties assessment tools of participation is important. We achieved that in Iran many researchers did not pay attention to the participation of CP children and many articles just paid attention to the sensory, motor or cognitive components of their clients. Among the life areas of participation the most focuses is on the ADL and social participation areas and the other areas are least important. Therefore, the suggestion of this study is that the researchers pay attention to the other areas of occupations and life areas of Iranian CP children because none of these areas are preferred over another.
